# 3D phytomer-based geometric modelling method for plants—the case of maize

**DOI:** 10.1093/aobpla/plab055

**Published:** 2021-09-08

**Authors:** Weiliang Wen, Yongjian Wang, Sheng Wu, Kai Liu, Shenghao Gu, Xinyu Guo

**Affiliations:** 1 Beijing Research Center for Information Technology in Agriculture, Shuguang Huayuan Middle Road, Haidian District, Beijing 100097, China; 2 Beijing Key Lab of Digital Plant, National Engineering Research Center for Information Technology in Agriculture, Shuguang Huayuan Middle Road, Haidian District, Beijing 100097, China

**Keywords:** Maize, morphology, three-dimensional modelling, three-dimensional phytomer, structure, visualization

## Abstract

Geometric plant modelling is crucial in *in silico* plants. Existing geometric modelling methods have focused on the topological structure and basic organ profiles, simplifying the morphological features. However, the models cannot effectively differentiate cultivars, limiting FSPM application in crop breeding and management. This study proposes a 3D phytomer-based geometric modelling method with maize (*Zea Mays*) as the representative plant. Specifically, conversion methods between skeleton and mesh models of 3D phytomer are specified. This study describes the geometric modelling of maize shoots and populations by assembling 3D phytomers. Results show that the method can quickly and efficiently construct 3D models of maize plants and populations, with the ability to show morphological, structural and functional differences among four representative cultivars. The method takes into account both the geometric modelling efficiency and 3D detail features to achieve automatic operation of geometric modelling through the standardized description of 3D phytomers. Therefore, this study provides a theoretical and technical basis for the research and application of *in silico* plants.

## Teaser

Geometric modelling of plants is very important for research and applications in agriculture, yet is challenging due to the complexity of plant architecture and morphology. In this paper, the authors proposed a 3D phytomer-based geometric modelling method for maize shoots. Phytomers, shoots, and populations were represented using simple mathematical symbols. 3D model of a maize shoot or population could be generated by assembling 3D phytomers and adjusting morphological parameters, just like assembling of industrial parts. This modelling method was efficient, and geometric models generated using the method were realistic.

## Introduction

Plant morphology reflects gene expression, resource acquisition and reproduction in plants. Plant morphology is also a direct consequence of the conditions and environment a plant grows in. Notably, integrating plant three-dimensional (3D) morphometrics is necessary for simulation and analysis of genetic cognition, adaptability evaluation and production capacity prediction (Bucksch *et al*. 2017; Gibbs *et al*. 2017; Marshall-Colon *et al*. 2017). Geometric plant models are key in computational studies related to Functional–Structural Plant Models (FSPMs) (Vos *et al*. 2010; Louarn and Song 2020), *in silico* plants (Marshall-Colon *et al*. 2017), plant phenomics (Paulus 2019; Zhao *et al*. 2019) and digital media content production focusing on agriculture. Researchers have raised 3D plant model construction standards for convenience and realism due to the rapid development of plant 3D data acquisition, computer graphics and computational performance.

The 3D model of a plant shoot is commonly built by assembling multiple organ models based on their topological relationship. The L-system approach proposed in 1968 is used to describe the assembly relationship between plant organs (Lindenmayer 1968). In early virtual plant research (Room *et al*. 1996), the 3D plant modelling is realized by integrating 3D digitized data acquisition, geometric modelling of organs and dynamic growth modelling of plants. Presently, L-studio (Prusinkiewicz *et al*. 1999), ADEL-Maize (Fournier and Andrieu 1999), GreenLab (Hu *et al*. 2003; de Reffye *et al*. 2020), OpenAlea (Pradal *et al*. 2008) and GroIMP (Hemmerling *et al*. 2013) are the commonly used plant modelling methods and software, which adopts the organ assemble strategy in 3D plant modelling. The strategy is convenient, efficient and easy to simulate and visualize complex structural plants, such as tree (Tondjo *et al*. 2018), cotton (Gu *et al*. 2014) and sunflower (Rey *et al*. 2008). However, since the constructed models do not adequately describe plant morphological features, such as wrinkles, twists and fold features in leaves that exist in different cultivars, they are mostly used for simulation and computational analysis among different species (Gaudio *et al*. 2019) and rarely used in crop breeding and management.

The 3D plant models are also built via 3D reconstruction from measured point cloud data through point cloud denoising, segmentation and reconstruction (Thapa *et al*. 2018; Zhu *et al*. 2020). The reconstructed 3D plant models are more realistic than the assembled modelling. However, 3D reconstruction algorithms with measured point cloud data must be developed towards specific species due to the structural differences and complex plant morphology. The 3D plant reconstruction has some limitations, such as 3D point cloud data acquisition (Wang *et al*. 2019), shoot to organ segmentation (Jin *et al*. 2019), feature-preserving organ reconstruction (Kempthorne *et al*. 2015) and organ mesh fusion (Yin *et al*. 2016). The solutions to these challenges are species-specific; hence manual interactions are needed in many operations. Therefore, it is difficult to develop a general and highly automatic 3D reconstruction of plant models.

The 3D design and assembling technology is widely used in the manufacturing field, forming a computer-aided manufacturing (CAM) model. Representative 3D design software for industrial parts, such as Pro-E and AutoCAD, has been used. The assemble procedure can be fully automated since the design and manufacture of each industrial part should be accurate and unambiguous. In contrast, the morphological structure and growth development of plants are caused by natural selection. Besides, most plants are flexible, thus difficult to realize the design and assembly as in industrial parts. Therefore, it is necessary to define and represent the basic plant unit in a 3D plant modelling based on the existing 3D data acquisition and geometric modelling technologies to achieve a fast, automatic and highly realistic assembling.

Cultivar-level morphological differences need to be captured in the current plant research and applications. For example, virtual cultivar selection for different eco-regions with specific environmental conditions could provide primary screening and reduce the experimental workload in the field. Also, new cultivar promotion via online visualization and interaction or virtual reality (VR) technologies could expand the product promotion channels for breeding companies. Therefore, convenient, accurate and realistic modelling methods are needed. In this study, the geometric modelling idea in computer-aided design (CAD) was introduced into 3D plant modelling to develop a novel approach based on 3D phytomers with maize as a representative plant. The geometric models of maize shoots and populations were constructed by defining and representing 3D phytomer digitally, considering efficiency and detail. This study provides a theoretical basis and practical technology for in-depth analysis and practical applications of *in silico* plants.

## Geometric Maize Modelling Based on 3D Phytomer

### Overview

**Figure 1.**
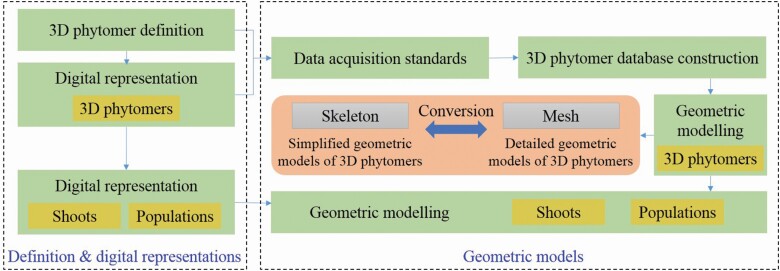
Geometric modelling workflow.

The geometric modelling method consists of two components (Fig. 1): (i) definition and digital representations, in which the 3D phytomers are defined and mathematical representations are given, and (ii) geometric models, involving data acquisition standards, 3D phytomer database construction and geometric modelling. The representation and geometric modelling are progressive in scales, from 3D phytomers to shoots, and finally to populations. In particular, conversion methods between simplified and detailed geometric models of 3D phytomers are also given to support the modelling process.

### Definition and digital representation of 3D phytomer

#### Definition of 3D phytomer for maize.

Phytomer refers to a functional unit of a plant that is repeatedly produced to form a shoot. It mainly consists of nodes, internodes, leaves and appendages. Phytomers differ between species because of the variations in structure and morphology among different plant species (de Reffye *et al*. 2020). In this study, maize phytomer refers to a combination of a node, an internode above the node and lateral organs grown on the node (Forster *et al*. 2007; McMaster and Hargreaves 2009).

**Figure 2.**
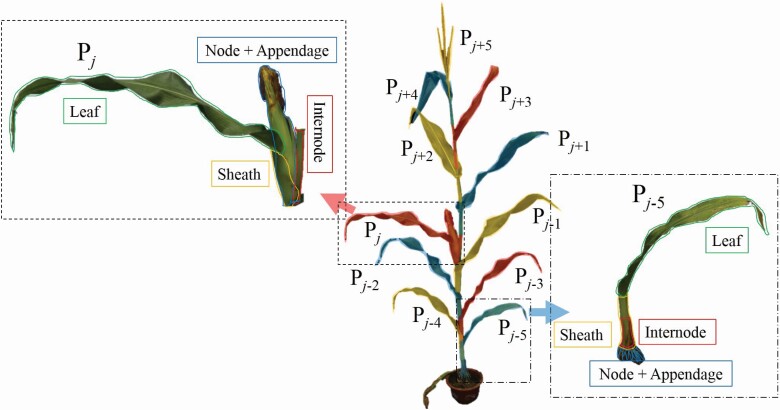
Definition of maize phytomer. A phytomer consists of a leaf (green wireframe), a sheath (yellow wireframe), an internode (red wireframe) enclosed by the sheath, a node and the appendage (blue wireframe) grown on the node. *P*_*j*_ is the ear phytomer, *P*_*j*–5_ is a nodal root phytomer and *P*_*j*+5_ is the tassel phytomer.

The maize shoot consists of several phytomers. *P*_*j*_ represents the *j*^th^ phytomer in a shoot, and the phytomer involves a node, a leaf, a sheath, an internode enclosed by the sheath and appendages grown on the node (Fig. 2). Tassel is the last phytomer on a maize shoot. It is a special phytomer because no leaf and sheath grow on it. There are two kinds of appendages, including axillary bud and nodal root. Notably, the maize ear is a grown-out bud that can be considered an appendage. The maize phytomers can be divided into five types based on the appendages and phytomer positions: root phytomer, ear phytomer, phytomer below the ear, phytomer above the ear and tassel phytomer. A maize shoot has several morphological units with a clear structure based on the phytomer definition. The phytomer was used to define 3D phytomer, a phytomer with spatial coordinate and morphological parameters, to realize 3D maize modelling assembly via the data acquisition approaches. Herein, 3D spatial coordinate information included two representation modes: complete mesh and simplified skeleton models.

#### Digital representation of 3D phytomer.

A digital representation of maize 3D phytomer was proposed based on its definition to quantitatively characterize maize 3D phytomers for subsequent modelling of maize shoots and populations:


$${\text{Phytomer}}_{j}=\left[M_{j},S_{j},Q_{j}\right]=\{\begin{array}{l} \left[M_{j}^{\text{Leaf}},S_{j}^{\text{Leaf}},Q_{j}^{\text{Leaf}}\right]\\ \left[M_{j}^{\text{Sheath}},S_{j}^{\text{Sheath}},Q_{j}^{\text{Sheath}}\right]\\ \left[M_{j}^{\text{Internode}},S_{j}^{\text{Internode}},Q_{j}^{\text{Internode}}\right]\\ \left[M_{j}^{\text{Appendage}},S_{j}^{\text{Appendage}},Q_{j}^{\text{Appendage}}\right] \end{array}$$ (1)

where $Q_{j}^{Leaf}=(H_{j}^{LeafBase},\;\;\;L_{j}^{Leaf},\;\;\;\theta_{j}^{Leaf},\;\;\;\alpha_{j}^{Leaf},\;\;\;\cdots ),$$Q_{j}^{sheath}=(H_{j}^{sheathBase},L_{j}^{sheath},\theta_{j}^{sheath},D_{j}^{sheathMax},D_{j}^{sheathMin}),$$Q_{j}^{Internode}=(H_{j}^{InternodeBase},L_{j}^{Internode},\theta_{j}^{Internode},D_{j}^{InternodeMax},D_{j}^{InternodeMin}),$ and $Q_{j}^{Appendage}=(D_{j}^{NodeMax},L_{j}^{Ear},D_{j}^{EarMax},...).$

Phytomer_*j*_ represents the *j*^th^ shoot phytomer, including the 3D mesh model *M*_*j*_, the skeleton model *S*_*j*_ and the morphological parameter set *Q*_*j*_. $M_{j}^{Leaf}$, $M_{j}^{Sheath}$, $M_{j}^{Internode}$ and $M_{j}^{Appendage}$ represent the 3D mesh model of leaf, sheath, internode and appendage on the *j*^th^ phytomer, respectively. The mesh models describe detailed spatial information of each component on a phytomer. Each mesh model contains all 3D point coordinates on a component and their relationships. $S_{j}^{Leaf}$, $S_{j}^{Sheath}$, $S_{j}^{Internode}$ and $S_{j}^{Appendage}$ represent the 3D skeleton model of the corresponding components. The skeleton models describe simplified spatial information of each component on a phytomer, such as a leaf vein and the central axis of an internode. Each skeleton model is described using a set of ordered 3D points, which is a subset of the vertices in the mesh model. $Q_{j}^{Leaf}$, $Q_{j}^{Sheath}$, $Q_{j}^{Internode}$ and $Q_{j}^{Appendage}$ represent corresponding morphological parameter set of the components on the *j*^th^ phytomer. The detailed parameters are outlined in **[see**Supporting Information—Table S1**]**. Each morphological parameter set is an extensible vector, and feature parameters can be added or deleted according to actual demands.

#### 3D phytomer resource database construction.

An accurate 3D mesh model, 3D skeleton model and morphological parameter set were used for maize geometric modelling by assembling the 3D phytomers. Therefore, a 3D digitizer was used to obtain maize phytomer templates with semantic information for 3D phytomer resource database construction. Construction and advancement of the cultivar-level database promote standardized storage, sharing and further application of 3D phytomers.

#### Data acquisition standards.

Standardized data acquisition is an important prerequisite for building an expandable database. The 3D digital data acquisition method described by Wang *et al*. (2019) was modified based on our 3D phytomer definition using the following standards. (i) 3D points characterizing a phytomer were obtained in rows from the junction of sheath and node to leaf tip. Five points were obtained in each row and only one point at the leaf tip. The fifth row was specified as the junction of a leaf and the corresponding sheath. The first and fifth points of each row were leaf margin points, and the third point in each row was vein point. (ii) The sheath and leaves were carefully removed after obtaining the sheath and leaf points. The points of the exposed internode were then obtained as in the case of the sheath. (iii) The points on each appendage were independently acquired. The three points, bottom, middle and top points of each branch on the tassel, were also determined. Several rows were determined from the bottom to the top of an ear, and five points were obtained in each row around the ear.

Each 3D coordinate obtained was semantic. For instance, leaf tip point, leaf margin points, leaf vein points, junction points between sheath and node were determined on a point set based on a specific order. Skeleton and mesh model of each phytomer were generated using the determined semantic points. The generated skeleton and mesh models were normalized to achieve upright internodes. The growth positions of the node in any phytomer were the point of origin. For instance $h_{i}^{Phytomer}$= 0 and the azimuth $\alpha_{i}^{Phytomer}$= 0. The standardized 3D phytomer templates were easily determined in subsequent geometry modelling.

#### 3D phytomer database construction.

A 3D phytomer database for maize was constructed using the data acquisition standards (Wen *et al*. 2017). There were four categories of keywords in the database: 3D mesh model information, corresponding morphological parameters, agronomic parameters of the node and other information (Table 1). Mesh models in the database were built using the acquired points, and the corresponding morphological parameters of each phytomer were extracted from the acquired points (Wen *et al*. 2018*a*) or through measurement. The agronomic parameters were manually recorded while the morphological parameters and other information were extensible items, and additional information was appended on according to actual needs.

**Table 1.** Keywords of maize 3D phytomer database

Type	Keywords
Agronomic parameters	Phytomer type, phytomer ID, cultivar, growth period, eco-region, density, row distance, water and fertilizer treatment
3D model template information	Storage path, name of phytomer model, vertices number, mesh number
Morphological parameter	Leaf length, leaf width, leaf azimuth, sheath length, sheath diameters, internode length, tassel length, tassel branch number, etc.
Other information	Template identifier, data acquisition time, data acquisition person, the person recording the data

### Geometric modelling of maize shoots and populations based on 3D phytomers

Geometric models of maize shoots and populations were constructed by integrating the digital representation and resource database of 3D phytomers.

#### Digital representation of maize shoots and populations.

Geometric maize shoot models combined several 3D phytomers by rotating, translating and assembling the selected 3D phytomers. The maize shoot digital representation was as follows:


$$\begin{array}{l} {Shoot}_{i}^{n}=U_{j=1}^{n}\;R_{ij}\cdot T_{ij}\cdot Phytomer_{ij} \end{array}$$ (2)

$Shoot_{i}^{n}$ indicates a maize shoot containing *n* expanded leaves, and *i* is the shoot index. $Phytomer_{ij}$ represents the *j*^th^ phytomer in the *i*^th^ shoot. $R_{ij}$ and $T_{ij}$ denote the rotation and translation matrix of the *j*^th^ phytomer in the *i*^th^ shoot.

Geometric maize population models were generated by rotating and translating several maize shoot models. Similarly, maize population was as follows:


$$\begin{array}{l} Population^{m}=\underset{i=1}{\overset{m}{U}}\;R_{i}\cdot T_{i}\cdot {Shoot}_{i}^{n}=\underset{i=1}{\overset{m}{U}}\;R_{i}\cdot T_{i}\cdot (\underset{j=1}{\overset{n}{U}}\;R_{ij}\cdot T_{ij}\cdot Phytomer_{ij}) \end{array}$$ (3)

$Population^{m}$ indicates a maize population containing *m* shoots. *R*_*i*_ and *T*_*i*_ are the rotation and translation matrix of the *i*^th^ shoot.

The direction of the constructed shoots and populations were vertical (the Z-axis was upright) since 3D phytomers were standardized. Therefore, the rotation matrices in the formula were in the positive direction of the Z-axis of the XY plane. Only the vertices coordinates in the mesh model of each 3D phytomer of maize shoot or population models are changed during translations. Besides, only the height-related parameters in *Q*_*j*_ are changed during the translation. However, both vertices coordinates in mesh model *M*_*j*_, and the azimuth-related parameters in *Q*_*j*_ are changed during rotations.

#### Geometric modelling of maize shoots and populations by assembling 3D phytomers.

The geometric modelling of maize shoots and populations was performed based on the resource database of 3D phytomer construction and digital representation. The target maize shoots and populations were constructed via two procedures. First, the 3D phytomer mesh models were selected by evaluating the similarity of morphological parameters. Second, the rotation and translation matrices in Equations (2) and (3) were determined.

**Figure 3.**
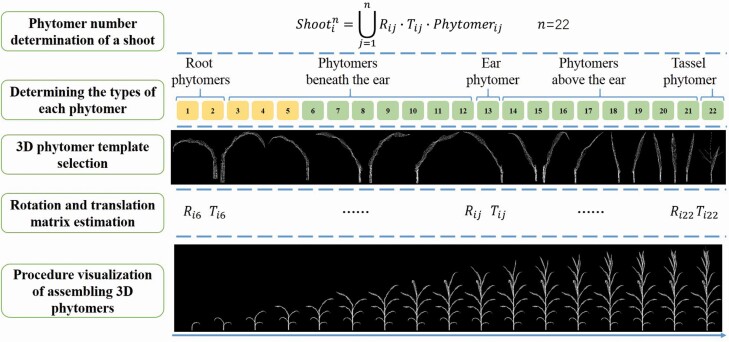
Illustration of maize shoot geometric modelling via assembling 3D phytomers. The process excluded the first to the fifth phytomer (yellow colored) because the leaves of these phytomers withered or fell.

The procedure of geometric modelling of maize shoots is illustrated in Fig. 3. First, the total phytomer number of the shoot was determined, and the type of each phytomer was specified. For instance, there were 22 phytomers of the shoot; first and second were root phytomers; the 3rd to the 12th were phytomers beneath the ear; the 13th was ear phytomer; the 14th to the 21st were phytomers above the ear; and the 22nd was the tassel phytomer (Fig. 3). The 3D phytomer templates were then selected from the resource database based on the phytomer type and highest similarity (Wen *et al*. 2018*b*). The origin point was set as the growth position of a new shoot model. The rotation matrix *R*_*ij*_ and the translation matrix *T*_*ij*_ were then calculated using the phytomer azimuth $\alpha_{i}^{Phytomer}$ and the phytomer height $h_{i}^{Phytomer}$, respectively. A geometric model of maize shoot was derived after determining parameters in Equation (2). The geometric modelling can be classified into two categories: 3D simulation (if the morphological parameters are specified by interactive design or derived by crop models) and 3D reconstruction (if derived by phenotype extraction from acquired image or point cloud data or directly measured).

Geometric models of shoots in the maize population were similar to the shoot modelling approach. In the population modelling, the shoot models were rotated and placed in the target population. The rotation matrix *R*_*i*_ and translation matrix *T*_*i*_ in Equation (3) were determined using the structural parameters of the target population, including total shoot number, density, row and planting distance, and the azimuth of each shoot in the population. The modelling is 3D simulation if the structural parameters are interactively designed or randomly generated, and 3D reconstruction if extracted from images or point clouds (Li *et al*. 2021) or *in situ* measured.

### Conversion between skeleton and mesh model

The skeleton and mesh models are simplified and detailed morphological representations of 3D phytomers. Semantic 3D phytomer data acquisition depends on 3D digitizer devices and is time-consuming. Acquiring point clouds of phytomers using 3D scanner or multi-view stereo (MVS) reconstruction is more applicable for most users. To obtain semantic mesh model from point clouds, we first extracted the skeleton from the coarse phytomer mesh, i.e. converting mesh to skeleton model. Next, we performed skeleton-driven deformation using the extracted skeleton and a semantic mesh model was derived, i.e. converting skeleton to mesh model. This is an alternative way of obtaining high-quality 3D phytomer templates and expands the usability of the modelling method. Therefore, conversion between the two models is essential for the 3D phytomer-based geometric modelling method. A leaf is an essential and complex organ in maize geometric modelling. Herein, a leaf was used as a representative organ to introduce the conversion method.

(1) Converting a mesh to skeleton model: There are two major methods used to obtain a leaf mesh model. The first method involves generating a leaf mesh using 3D digitized data. Each point acquired using 3D digitizers is ordered and semantic (Wen *et al*. 2018*a*). Since the vein and margin points are known, all the vein points can be selected through the point acquisition order, achieving mesh to skeleton conversion. The second method involves leaf mesh reconstruction from point cloud data. Here, points are disordered. Therefore, skeleton extraction algorithms are conducted to extract the skeleton points. In this study, an improved Laplacian-based skeleton extraction algorithm (Cao *et al*. 2010; Wu *et al*. 2019) was used to realize mesh to skeleton conversion.
(2) Converting skeleton to mesh model: Several continuous and ordered points represented leaf skeleton. A leaf mesh model with known vein points (e.g. the leaf mesh model generated using 3D digitized data) and minimum morphological differences with the target skeleton to be converted was searched in the 3D template database. The searched template mesh was scaled, rotated and transformed using the target skeleton. The target skeleton was then resampled based on the number of vein points in the leaf template to realize one-to-one correspondence between the skeleton and vein points in the template mesh. Finally, as-rigid-as-possible (ARAP) mesh deformation algorithm (Sorkine and Alexa 2007) was used to achieve skeleton-driven leaf mesh deformation, converting the target skeleton to a leaf mesh model.

## Materials and Methods

### Experiment design and data acquisition

The field experiment was conducted in 2018 at the experimental field of Beijing Academy of Agriculture and Forestry Sciences (39°56′ N, 116°16′ E), to evaluate the performance of the proposed geometric modelling method. Four cultivars, including Aidan268 (AD268), Demeiya2 (DMY2), Jingke968 (JK968) and Zhengdan958 (ZD958), were selected and planted on 4th June at six plants per square metre, and row distance of 60 cm. Three shoots of each cultivar with representative morphometrics were selected at each growth stage (V6, V9, V13 and R1) for evaluation (Abendroth *et al*. 2011). A high-throughput phenotyping platform MVS-Pheno was used to obtain multi-view images of the selected shoots, and 3D point clouds were reconstructed (Wu *et al*. 2020). Morphological parameters were then obtained through skeleton extraction (Wu *et al*. 2019), and used to construct the maize shoot and population models. The phytomer number of each shoot was then determined. Fastrak 3D digitizer (Polhemus, Colchester, VT, USA) was used following the data acquisition standards to obtain 3D template data of all shoot phytomers, including skeletons and meshes. The templates were then added to the resource database. In addition, the leaf length and width of all the shoot phytomers were measured to validate the results.

### Leaf area measurement and light interception estimation within maize canopy

Leaf areas were measured for accuracy evaluation. Because it is challenging to obtain accurate leaf area measurements, especially for complicated leaf shapes, such as leaves with many wrinkled features, we used a traditional method to estimate the leaf area: multiply leaf length by leaf width by 0.75 (Montgomery 1911).

The modelling approach and light simulation algorithm was applied and evaluated in our previous study with satisfactory accuracy (Wen *et al*. 2019*a*). This study used the proposed method to build canopies of four maize cultivars at the grain filling stage. Leaf index, leaf length and leaf angle were the primary phytomer parameters. Each canopy included 81 shoots with nine rows and nine shoots in each row. The 3 × 3 shoots lying in the centre of each canopy were selected to analyze the light interception differences among the cultivars. The light simulation experiment was conducted from 7 am to 5 pm on 30 June 2019. Total intercepted photosynthetically active radiation (PAR) of each phytomer was accumulated within the experimental period.

## Results

### Geometric models of maize shoots and populations constructed using 3D phytomers

#### Geometric modelling results of maize shoots.

**Figure 4.**
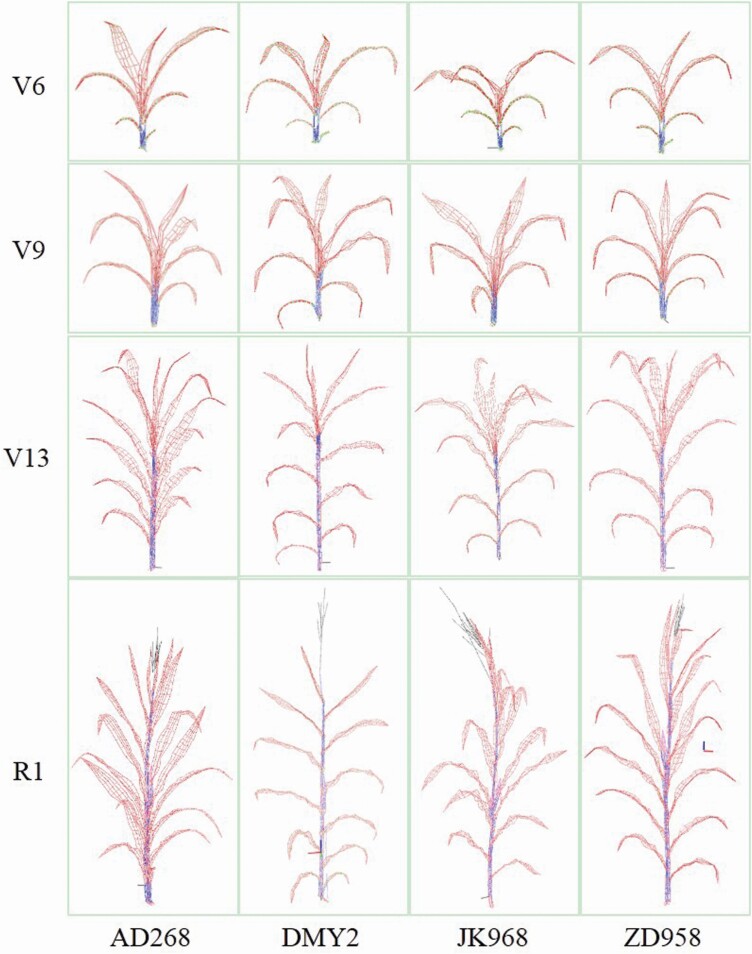
Visualization of maize shoot models of four cultivars at four growth stages using 3D phytomer-based modelling method. The y-axis labels V6, V9, V13 and R1 refer to 6th leaf, 9th leaf, 13th leaf, and silking stage, respectively.

The proposed geometric modelling method was used to construct models of four cultivars at four growth stages. The cultivars had significant morphological differences at various growth stages (Fig. 4). The plant height and interval between adjacent phytomers of AD268 were shorter than other cultivars, while the leaf widths were larger. DMY2 leaves were long and narrow, and the shoots were relatively flat. The lower JK968 leaves were flatter, and the height intervals of adjacent phytomers near the ear were short. However, the intervals between phytomers away from the ear were long. The upper ZD958 leaves were smaller and more compact than other cultivars. Besides, the ZD958 plants had similar height intervals.

**Figure 5.**
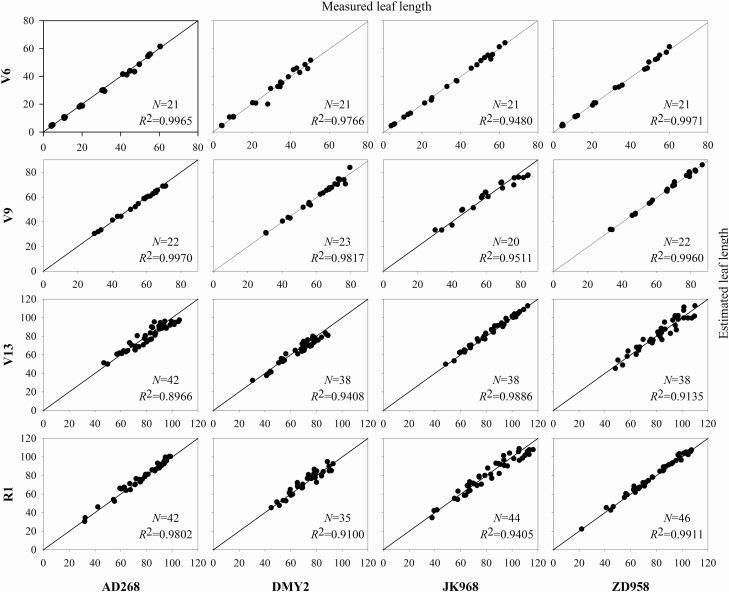
A comparison between the estimated and measured leaf length of four cultivars at four growth stages.

**Figure 6.**
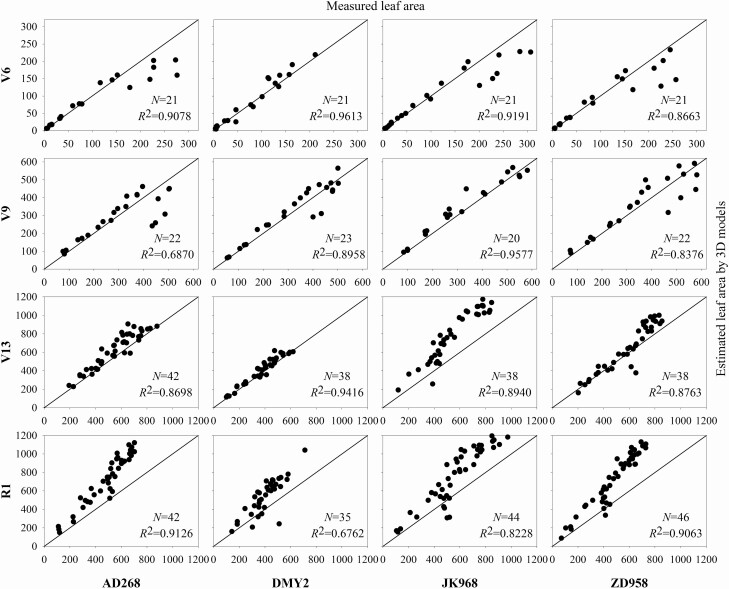
A comparison between the estimated and measured leaf area of four cultivars at four growth stages. The leaf area was estimated using the formula: measured leaf length multiplied by leaf width by 0.75.

To evaluate the modelling accuracy, the estimated leaf length and area of each phytomer were compared with corresponding measurements. A comparison between the measured and estimated the leaf length and leaf area of four cultivars at four growth stages is shown in Figs 5 and 6. The estimated leaf length did not differ significantly from the measured ones, and the average *R*^2^ was 0.9628. In contrast, the estimated leaf area differed considerably from the measured ones with an averaged *R*^2^ of 0.8708, especially for late growth stage shoots. The estimated leaf area is usually larger than the measured one. This is because the traditional leaf area measurement method does not consider the leaf shape diversity among different cultivars. In comparison, 3D models consider the morphological features of different cultivars since featured points can be obtained during data acquisition. Therefore, geometric models constructed using this method are more reliable. Similar findings were reported in our previous study (Wang *et al*. 2019).

#### Geometric modelling of maize populations.

**Figure 7.**
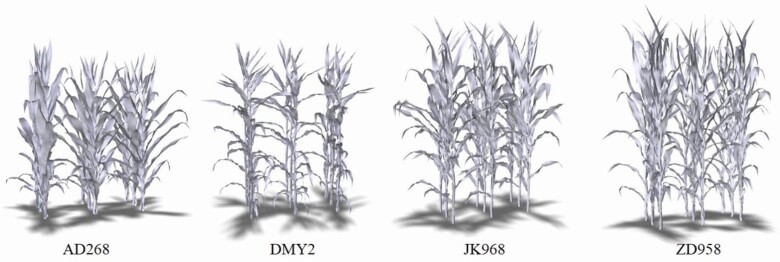
Visualization of four maize population models generated via 3D phytomer-based modelling method.

 The geometric maize population model was constructed based on the 3D maize shoot models and population parameters. Population models of the four cultivars before tasseling are shown in Fig. 7. The structural modelling parameters were similar in the four models. The row was from south to north with a plant spacing of 60 and 27.8 cm, respectively. The geometric model constructed using this method can be used for FSPM studies, such as calculating light distribution within maize canopies (Wen *et al*. 2019*a*). In addition, a phytomer scale can be used to analyze the calculation results.

#### Modelling efficiency.

Similarity calculation to select 3D phytomer template, rotation and translation of selected phytomers are relatively the most time-consuming procedures during shoot modelling. However, the matching calculation is relatively fast since similarity calculation matches 3D phytomers in a specific cultivar sub-database. Moreover, rotation and translation are fast since a 3D phytomer template contains less than 200 vertices. Therefore, the maize shoot model can be constructed in real time. Geometric maize population modelling is realized by constructing multiple geometric models of maize shoots, translation and rotation. Therefore, the construction efficiency is directly related to the number of shoots in the population. Practically, modelling a maize population with 200 shoots can be achieved within two seconds. The 3D phytomer data are obtained based on given standards and are classified and stored using keywords, such as cultivar. Therefore, the 3D phytomer-based modelling method is efficient and convenient for data acquisition, management, and use.

#### 3D phytomer conversion between skeleton and mesh models.

**Figure 8.**
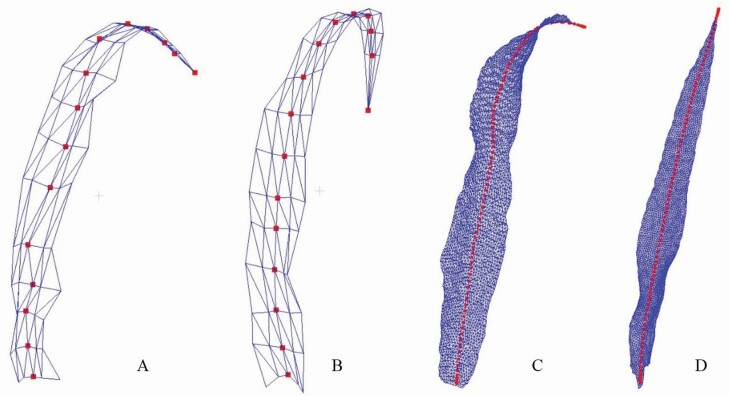
Visualization of mesh to skeleton conversion of 3D phytomers. (A, B) Conversion from leaf mesh generated via 3D digitized data. (C, D) Conversion from leaf mesh generated using 3D point cloud data.

The above geometric modelling results show the performance of the proposed method on shoot and population scale based on 3D phytomers. Here, the conversion results between the skeleton and mesh models within the phytomer scale are presented. Conversion results from leaf mesh to skeleton were based on data source. The mesh to skeleton model conversion results generated using 3D digitizer acquired data are shown in Fig. 8A and B. The 3D phytomer data acquired had clear semantic information and could directly retrieve the vein points. However, it depends on the manual data acquisition accuracy. The conversion results from mesh generated via dense 3D point clouds are shown in Fig. 8C and D. This conversion is automatic, and its accuracy depends on the skeleton extraction algorithms (Wu *et al*. 2019).

**Figure 9.**
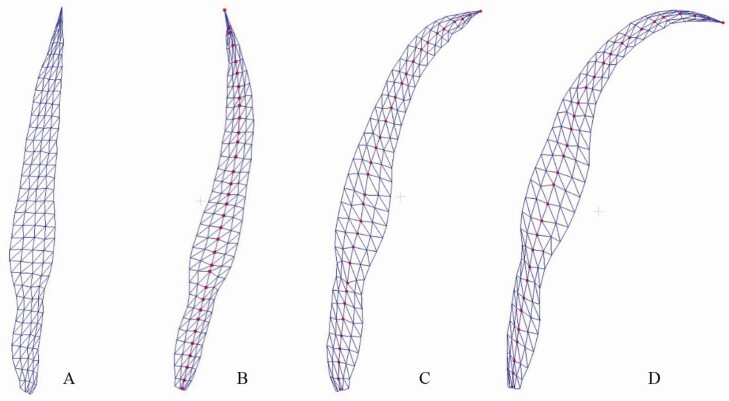
Visualization of the skeleton to mesh conversion of 3D phytomers. (A) Leaf mesh model. (B–D) Three conversion results using different skeletons as inputs. The red points in B–D indicate the skeleton.

The maize leaves were selected to evaluate conversion methods between skeleton and mesh models. The conversion results from skeleton to mesh models are shown in Fig. 9. Leaf shape was maintained after the skeleton-driven deformation algorithm. Leaf skeletons were obtained through interactive design, skeleton extraction from point clouds, or using 3D digitizers. Therefore, combining the leaf templates of various cultivars, with the skeleton to mesh conversion method facilitates efficient mesh generation and decreases data acquisition workload. Furthermore, the generated 3D phytomer mesh models were realistic.

### Analysis of phytomer scale light interception

**Figure 10.**
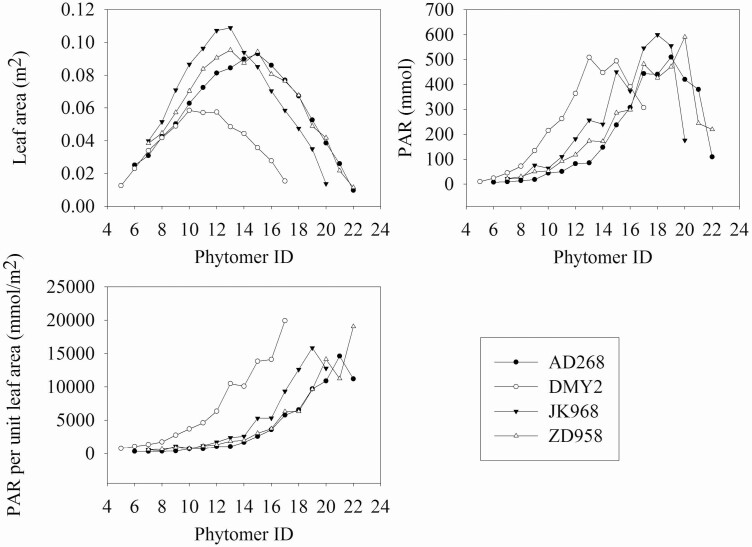
The average leaf area, intercepted PAR, and intercepted PAR per leaf area of the four cultivars.

Light interception analysis in phytomer scale was conducted to test whether it is possible to present differences among cultivars. Figure 10 shows the average leaf area, intercepted PAR and intercepted PAR per leaf area of the four cultivars. There was no significant difference in leaf area of lower phytomers between the four cultivars. DMY2 was the smallest and JK968 was the largest in middle phytomers; AD268 and ZD958 had the largest leaf area of upper phytomers. The total intercepted PAR of lower and middle phytomers of DMY2 was significantly higher than that of the other cultivars. This is because the PAR was less sheltered by the upper leaves. The total intercepted PAR of middle and upper phytomers was significantly higher in DMY2 than in AD268 and ZD958, suggesting fewer phytomers, and the leaves were more spatially dispersed to intercept more light. DMY2 exhibited significantly higher intercepted PAR per leaf area than other cultivars. The intercepted PAR per leaf area of JK968 at middle phytomers was higher than that of AD268 and ZD958. The middle phytomers were the main dry matter synthesis areas at the grain filling stage, which potentially enabled JK968 to produce more grain dry matter. These findings demonstrate that the proposed method can be used for 3D modelling and FSPM analysis at the phytomer scale.

## Discussion

### Advantages of the 3D phytomer-based modelling method

This study proposed the digital 3D phytomer representation, an advancement of the traditional phytomer (Sattler and Rutishauser 1997). Besides topological structure, the digital representation can also quantitatively describe the morphological features, improving the 3D plant research from structure to morphology. The proposed modelling method has the following advantages:

(1) The modelling method proposed in this paper improves the assembling units from organs to 3D phytomer scale. The maize shoot assembling is realized via direct translation and rotation of 3D phytomers (Fig. 3), without the fusion details. Therefore, it is more suitable for users with no computer graphic knowledge, has better universality and is realistic. In contrast, existing methods are convenient in describing the topological structure and morphological details of a single organ, they are not suitable for detailed whole plant assembling. For example, assembling leaf and sheath or sheath and internode is not realistic at the connection areas (He *et al*. 2021). Mesh fusion in computer graphics is also challenging (Yin *et al*. 2016). It is also difficult to improve the accuracy and detail of 3D plant models, limiting the practicability of modelling model or software.
(2) 3D phytomers have clear semantic details, such as organ types, morphological parameters, connections and spatial coordinates. Therefore, researchers can identify the detailed information of interest in the phytomer, shoot and population scales, thus effective and convenient (Wen *et al*. 2018*b*). Comparatively, the formal grammars were used to generate 3D plant models and growth processes in process-based modelling methods, such as L-system (Prusinkiewicz *et al*. 1999). However, it is difficult to extract or understand specific morphological parameters and spatial positions in the model through string expressions (Hu *et al*. 2003).
(3) The whole modelling process is very simple and efficient because maize shoots or populations can be directly written as unions of rotation and translation of multiple phytomers (Fig. 3). The 3D phytomers are the standard parts in the assembled plant modelling. The standard parts have clear semantic information that can be applied in the plant model construction. Furthermore, the software can be automated (Lu *et al*. 2014). Omics research and gene editing can be considered as the extraction and editing of the internal components of standard plant parts (Shakoor *et al*. 2019). The 3D phytomer promotes the transformation of digital plant research to CAM mode. Therefore, this study provides a basis for the transformation process.
(4) The proposed method can improve the resolution of plant organ features (Fig. 9) while ensuring the accuracy of plant topology and improving subsequent visual computation accuracy (Wen *et al*. 2015). For instance, existing plant modelling software or model requires more complex operations to generate 3D leaf models with a fold. However, the folding feature is associated with the leaf direction, directly impacting canopy light distribution calculation (Kim *et al*. 2020). Besides, 3D leaf features of different cultivars are associated with leaf area differences (Fig. 6). These small leaf area variations can enlarge the accumulated error in the final results when estimating accumulated PAR interception within a specific time interval. Thus, it is challenging to find the detailed cultivar differences regarding light interception. Collectively, these findings show that the 3D phytomer-based modelling method can improve the accuracy of geometric modelling-related research and applications.
(5) In addition to the topological structure, 3D morphometrics is added to phytomers via the mesh representation, which poses greater challenges for the proposed method to deal with the dynamic growth modelling (Guo *et al*. 2006). However, the 3D morphological information enables the method to achieve higher resolution simulation of dynamic plant changes, such as simulating the daily changes of plant leaves and the 3D shape changes of plants due to water loss wilting (Zhang *et al*. 2017). This advancement enables researchers to better understand and replicate the plant stress due to environmental factors, which is difficult to achieve using the topological structure-based modelling approaches.

However, the proposed method has some limitations. First, it is time-consuming and labour-intensive during data acquisition to ensure the data quality of 3D phytomers. The 3D phytomer database must practically obtain certain data (different growth periods, cultivars, densities and eco-region data). For instance, the modelling method is not efficient when it has insufficient data. Similarly, deep learning requires a large amount of data for model training to improve the automatic level in subsequent procedures (Griffiths and Boehm 2019).

Maize was selected to demonstrate the modelling method in this study because it exhibits a large structure with no branches. The proposed method is suitable for analyzing cereal crops with known phytomer characteristics. For instance, a wheat or rice shoot can be divided into stem and tillers, which can be further decomposed into several phytomers. Thus, the method, including digital representation of 3D phytomer, data acquisition standards, geometric modelling of stem or tiller, shoot, and population, can be extended and applied in these cereal plants. However, the method is unsuitable for fruit trees, capsicum and other plants with complex branching structures. This has limited phytomer classification in these plants.

### Combination of 3D data acquisition and plant phenomics with the proposed method

The 3D phytomer-based modelling method can be easily integrated with multiple 3D data acquisition approaches (Wen *et al*. 2019*b*). Besides digitization data, point clouds of phytomers acquired using 3D scanning or multi-view stereo reconstruction can be used to construct 3D phytomer templates (Figs 8 and 9). A semantic mesh model can be practically generated through feature extraction and surface reconstruction or using software to select the feature points. The generated 3D phytomer templates can identify cultivar differences (Fig. 4) and can be used for subsequent visual computation of crop breeding and management since *in situ* measured data contain detailed characteristics (Wen *et al*. 2017).

Crop Phenomics (Yang *et al*. 2020) is currently a research hotspot in crop science. Crop phenotyping focuses on identifying phenotypic traits from 3D point cloud data (Jin *et al*. 2021) to realize quantitative description or measurement of structural and morphological features of plants. Furthermore, constructing plant geometric models using the extracted structural and morphological parameters can improve the understanding of plant phenomics. For instance, using high-throughput phenotyping platforms to obtain images (Cabrera-Bosquet *et al*. 2016) or point clouds (Wu *et al*. 2020) of maize shoots can build 3D plant models, thus achieving canopy light distribution analysis of different cultivars. However, 3D plant model developed using such methods is not suitable for high-resolution studies. In contrast, efficient and realistic data-driven geometric maize shoot modelling can be achieved by integrating extracted phenotypic parameters combined with this 3D phytomer-based plant modelling method.

### Potential applications and future work

The geometric modelling method can be used to conduct virtual experiments for different eco-regions and cultivars, providing theoretical guidance for crop management studies due to the 3D phytomer database advancement and data volume increase. The 3D phytomer database contains structural and morphological data of various cultivars obtained from different environmental conditions. Besides, the data are highly structuralized 3D scale data with semantic information. Therefore, further studies can be conducted using the database, combined with knowledge mapping and artificial intelligence technologies. Moreover, 3D maize models constructed using this method are realistic and can be used in digital media content production focusing on agriculture. The method has been presented as the underlying engine in PlantCAD (Lu *et al*. 2014), an interactive 3D modelling software for plants. However, further studies are necessary to provide high-quality 3D phytomer data following the data acquisition standards, enrich the database and upgrade the modelling software.

## Supporting Information

The following additional information is available in the online version of this article—

**Table S1.** Detailed parameter definition and description of 3D phytomer. The 3D phytomer database including the 3D phytomers of four cultivars was also provided.

plab055_suppl_Supplementary_Data

plab055_suppl_Supplementary_Table_S1

## Sources of Funding

This work was partially supported by the National Natural Science Foundation of China (31871519, 32071891), Science and Technology Innovation Special Construction Funded Program of Beijing Academy of Agriculture and Forestry Sciences (KJCX20210413), Reform and Development Project of Beijing Academy of Agricultural and Forestry Sciences, and China Agriculture Research System of Ministry of Finance and Ministry of Agriculture and Rural Affairs.

## Conflict of Interest

None declared.

## Contributions by the Authors

Conceptualization, X.G.; methodology, W.W. and Y.W.; software, W.W., S.W. and K.L.; validation, Y.W.; data curation, Y.W.; writing-original draft preparation, Y.W., W.W. and X.G.; writing-review and editing, W.W. and Y.W.; visualization, S.G.; supervision, X.G.; project administration and funding acquisition, X.G. and W.W.

## Notes

**Form & Function. Chief Editor**: Kate McCulloh
