# Supplementary material for: 3D phytomer-based geometric modelling method for plants—the case of maize
Source: AoB Plants. 2021 Sep 8;13(5):plab055. doi: 10.1093/aobpla/plab055 (PMC8482417; doi:10.1093/aobpla/plab055)
Supplement: plab055_suppl_Supplementary_Table_S1 [file plab055_suppl_supplementary_table_s1.docx]

Table S1. Detailed parameter definition and description of 3D phytomer

| Classification | Abbreviation | Parameter | Description |
| --- | --- | --- | --- |
| Whole phytomer | $h_{i}^{Phytomer}$ | Phytomer height | Vertical distance from the lowest point of the phytomer to ground. |
|  | $\alpha_{i}^{Phytomer}$ | Phytomer azimuth |  |
| Leaf | $h_{i}^{LeafBase}$ | Leaf base height | Vertical distance from leaf base to ground |
|  | $h_{i}^{LeafTop}$ | Leaf top height | Vertical distance from the highest point to ground |
|  | $h_{i}^{LeafTip}$ | Leaf tip height | Vertical distance from leaf tip to ground |
|  | $l_{i}^{Leaf}$ | Leaf length |  |
|  | $w_{i}^{Leaf}$ | Leaf width |  |
|  | $\theta_{i}^{Leaf}$ | Leaf angle |  |
|  | $\alpha_{i}^{Leaf}$ | Leaf azimuth |  |
|  | $s_{i}^{Leaf}$ | Leaf area |  |
| Sheath | $h_{i}^{SheathBase}$ | Sheath base height | Vertical distance from sheath base to ground |
|  | $l_{i}^{Sheath}$ | Sheath length |  |
|  | $\theta_{i}^{Sheath}$ | Sheath angle | Sheath inclination |
|  | $d_{i}^{SheathMax}$ | Sheath maximum diameter |  |
|  | $d_{i}^{SheathMin}$ | Sheath minimum diameter |  |
| Internode | $h_{i}^{InternodeBase}$ | Internode base height | $h_{i}^{InternodeBase}$= $h_{i}^{SheathBase}$。 |
|  | $l_{i}^{Internode}$ | Internode length |  |
|  | $\theta_{i}^{Internode}$ | Internode angle | Internode inclination |
|  | $d_{i}^{InternodeMax}$ | Internode maximum diameter |  |
|  | $d_{i}^{InternodeMin}$ | Internode minimum diameter |  |
| Tassel | $l_{i}^{Tassel}$ | Tassel length | Main branch length of the tassel |
|  | $n_{i}^{Tassel}$ | Tassel branch number |  |
|  | $\theta_{i}^{Tassel}$ | Tassel branch angle | Averaged inclination of all tassel branches |
| Appendage | $d_{i}^{NodeMax}$ | Node maximum diameter |  |
|  | $d_{i}^{NodeMin}$ | Node minimum diameter |  |
|  | $l_{i}^{Ear}$ | Ear length |  |
|  | $d_{i}^{EarMax}$ | Ear maximum diameter |  |
|  | $n_{i}^{NodalRoot}$ | Nodal root number |  |
|  | $d_{i}^{NodalRoot}$ | Averaged diameter of nodal roots |  |
